# Supplementary material for: Effects on Pinus densiflora Seedlings as Affected by Different Container Growth Conditions
Source: Int J Environ Res Public Health. 2020 May 19;17(10):3565. doi: 10.3390/ijerph17103565 (PMC7277831; doi:10.3390/ijerph17103565)
Supplement: Supplementary file 1 [file ijerph-17-03565-s001.pdf]

Supplementary Figure

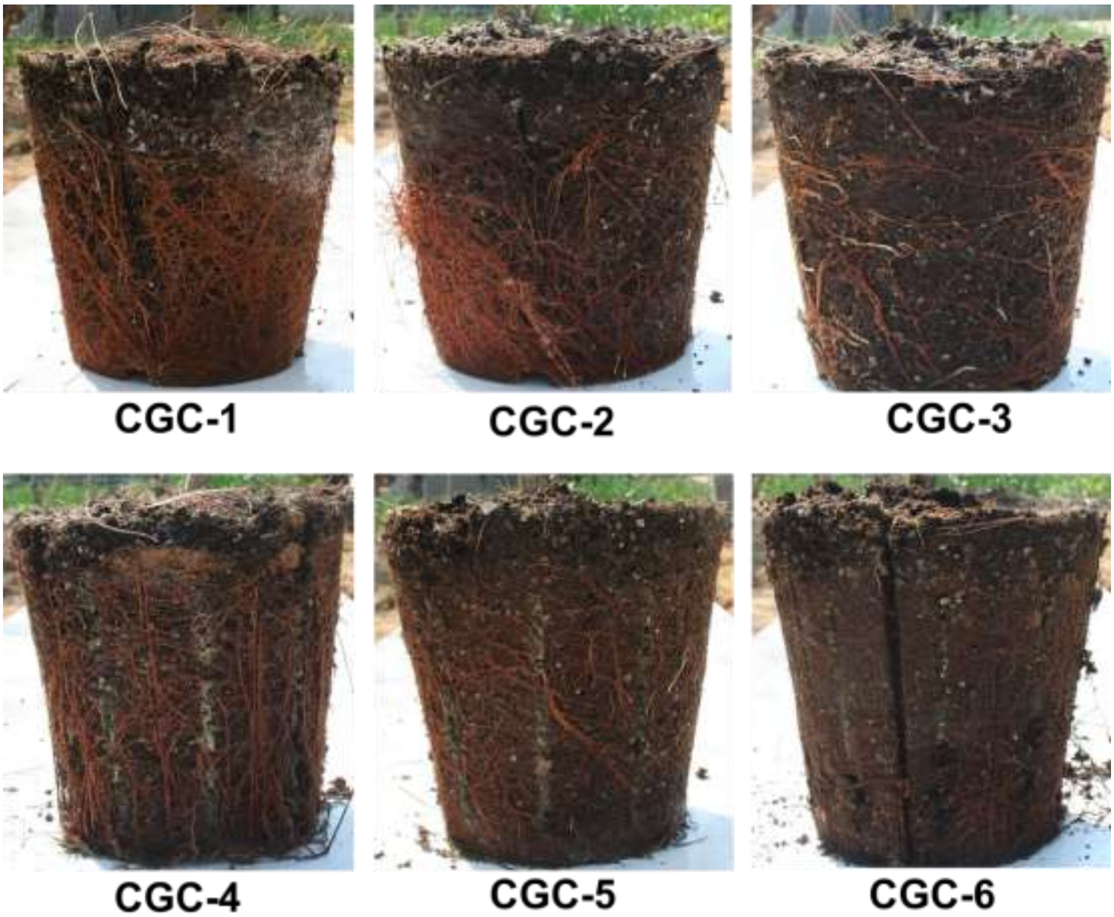

Figure 1. *Pinus densiflora* seedling root status affected by six different container growth conditions (CGCs).
